# Polygenic Models Partially Predict Muscle Size and Strength but Not Low Muscle Mass in Older Women

**DOI:** 10.3390/genes13060982

**Published:** 2022-05-30

**Authors:** Praval Khanal, Christopher I. Morse, Lingxiao He, Adam J. Herbert, Gladys L. Onambélé-Pearson, Hans Degens, Martine Thomis, Alun G. Williams, Georgina K. Stebbings

**Affiliations:** 1 Musculoskeletal Science and Sports Medicine Research Centre, Department of Sport and Exercise Sciences, Manchester Metropolitan University Institute of Sport, Manchester Metropolitan University, Manchester M15 6BH, UK; c.morse@mmu.ac.uk (C.I.M.); lingxiao.he@hotmail.com (L.H.); g.pearson@mmu.ac.uk (G.L.O.-P.); a.g.williams@mmu.ac.uk (A.G.W.); g.stebbings@mmu.ac.uk (G.K.S.); 2 Physical Activity, Sports & Health Research Group, Department of Movement Sciences, KU Leuven, 3001 Leuven, Belgium; martine.thomis@kuleuven.be; 3 Department of Medical Biochemistry, Nobel College, Affiliated to Pokhara University, Kathmandu 44600, Nepal; 4 Research Centre for Life and Sport Sciences (CLaSS), Department of Sport and Exercise, School of Health Sciences, Birmingham City University, Birmingham B5 5JU, UK; adam.herbert@bcu.ac.uk; 5 Musculoskeletal Science and Sports Medicine Research Centre, Department of Life Sciences, Manchester Metropolitan University Institute of Sport, Manchester Metropolitan University, Manchester M15 6BH, UK; h.degens@mmu.ac.uk; 6 Institute of Sport Science and Innovations, Lithuanian Sports University, LT-44221 Kaunas, Lithuania; 7 Institute of Sport, Exercise and Health, University College London, London W1T 7HA, UK; 8 Applied Sports, Technology, Exercise and Medicine Research Centre (A-STEM), College of Engineering, Swansea University, Swansea SA2 8PP, UK

**Keywords:** polygenic model, predisposing allele, skeletal muscle phenotypes, low and high muscle mass

## Abstract

Background: Heritability explains 45-82% of muscle mass and strength variation, yet polygenic models for muscle phenotypes in older women are scarce. Therefore, the objective of the present study was to (1) assess if total genotype predisposition score (GPS_TOTAL_) for a set of polymorphisms differed between older women with low and high muscle mass, and (2) utilise a data-driven GPS (GPS_DD_) to predict the variance in muscle size and strength-related phenotypes. Methods: In three-hundred 60- to 91-year-old Caucasian women (70.7 ± 5.7 years), skeletal muscle mass, biceps brachii thickness, vastus lateralis anatomical cross-sectional area (VL_ACSA_), hand grip strength (HGS), and elbow flexion (MVC_EF_) and knee extension (MVC_KE_) maximum voluntary contraction were measured. Participants were classified as having low muscle mass if the skeletal muscle index (SMI) < 6.76 kg/m^2^ or relative skeletal muscle mass (%SMM_r_) < 22.1%. Genotyping was completed for 24 single-nucleotide polymorphisms (SNPs). GPS_TOTAL_ was calculated from 23 SNPs and compared between the low and high muscle mass groups. A GPS_DD_ was performed to identify the association of SNPs with other skeletal muscle phenotypes. Results: There was no significant difference in GPS_TOTAL_ between low and high muscle mass groups, irrespective of classification based on SMI or %SMM_r_. The GPS_DD_ model, using 23 selected SNPs, revealed that 13 SNPs were associated with at least one skeletal muscle phenotype: *HIF1A* rs11549465 was associated with four phenotypes and, in descending number of phenotype associations, *ACE* rs4341 with three; *PTK2* rs7460 and *CNTFR* rs2070802 with two; and *MTHFR* rs17421511, *ACVR1B* rs10783485, *CNTF* rs1800169, *MTHFR* rs1801131, *MTHFR* rs1537516, *TRHR* rs7832552, *MSTN* rs1805086, *COL1A1* rs1800012, and *FTO* rs9939609 with one phenotype. The GPS_DD_ with age included as a predictor variable explained 1.7% variance of biceps brachii thickness, 12.5% of VL_ACSA_, 19.0% of HGS, 8.2% of MVC_EF_, and 9.6% of MVC_KE_. Conclusions: In older women, GPS_TOTAL_ did not differ between low and high muscle mass groups. However, GPS_DD_ was associated with muscle size and strength phenotypes. Further advancement of polygenic models to understand skeletal muscle function during ageing might become useful in targeting interventions towards older adults most likely to lose physical independence.

## 1. Introduction

Skeletal muscle phenotypes, particularly muscle mass and muscle strength, are determined by multiple factors [1,2], and it has been reported that heritability may explain 45–82% of these phenotypes [3,4,5]. Over 200 gene variants have been linked with health-related fitness phenotypes [6], and many cross-sectional and longitudinal studies have identified single-nucleotide polymorphisms (SNPs) that are associated with skeletal muscle phenotypes, specifically muscle mass and strength [6,7,8,9]. Yet, only a limited number of these SNPs, such as *ACTN3* and *ACE,* have shown consistent associations with skeletal muscle phenotypes in different populations, while most SNPs have been investigated only once or have shown contradictory results [10,11]. Perhaps part of the problem is that there is considerable (16–20%) inter-individual variability in skeletal muscle mass and strength [12] that is under polygenic control, contributing to the lack of successful replications between these phenotypes and single gene variants [10]. Attempts to explain some of the variability in these phenotypes via single SNP analyses has had some success. *ACTN3* R577X, for example, explained 1–1.4% of the variation in knee extension torque [13], but this was less than the 3–9% of variation explained when using a polygenic approach in male coronary artery disease patients [14]. Therefore, the inclusion of multiple gene variants that may influence skeletal muscle phenotypes might explain a greater proportion of the variance observed within skeletal muscle phenotypes. An understanding of the polygenic profile of skeletal muscle phenotypes among the older population may be beneficial for the understanding of the development and risk of disability and sarcopenia in old age [15].

Given that women have a lower muscle mass than men and hence any loss of muscle mass will cause them to cross a disability threshold earlier, it is no surprise that women experience more severe loss of mobility as a result [16]. Considering the importance of muscle mass/strength for maintaining mobility [17], reducing length of hospital stay [18], and delaying mortality during ageing [19], it is necessary to investigate the polygenic influence on important skeletal muscle phenotypes, such as muscle size and strength, in an older population. Below established thresholds of either skeletal muscle mass relative to body mass (%SMM_r_), or skeletal muscle mass relative to height^2^, termed the skeletal muscle index (SMI), older individuals can be defined as having low muscle mass equivalent to “pre-sarcopenia” [20,21]. The pre-sarcopenia thresholds for both muscle mass indices have been established to indicate adverse outcomes associated with ageing. For instance, a %SMM_r_ < 22.1% has been linked to higher risk of functional impairment and physical disability in older women [20], while an SMI < 6.76 kg/m^2^ in older women was associated with elevated risk of physical disability [21]. Given the potential for even independently living older women to fall under this pre-sarcopenic classification of low muscle mass, understanding the polygenic influence on skeletal muscle phenotypes may allow us to identify those at increased risk of experiencing low muscle mass with ageing.

The polygenic concept was first introduced to explore the genetic contribution to elite human performance in hypothetical populations [22], and this approach has been later successful in distinguishing elite athletes in strength-related sports from non-athlete populations [23,24,25,26]. Over time, different approaches have been developed to study the polygenic influence on muscle, but the basis of each approach is to allocate a genotype score (GS) according to the assumed positive/negative effect of alleles on phenotypes [27]. The GPS_DD_ is one example of how the polygenic approach has changed [14,28,29]. GPS_DD_ differs from the GPS_TOTAL_ by only including SNPs that were significantly associated with the phenotype(s) of interest in the analysis. In addition, the incorporation of other factors (such as age, sex, diet, and physical activity) in GPS_DD_ may explain more of the variance in skeletal muscle phenotypes [27].

Recently, our group has identified a set of SNPs related to skeletal muscle phenotypes and low muscle mass using the single candidate gene approach in older women [30,31]. Yet, the cumulative effect of SNPs on ageing muscle phenotypes may be better understood if a polygenic model is adopted. Furthermore, no study has assessed whether there is a difference in GPS_TOTAL_ between a low muscle mass group (equivalent to pre-sarcopenia thresholds of SMI and %SMM_r_) and a high muscle mass group. The objective of the present study was, therefore, to (1) compare a GPS_TOTAL_ (including 23 SNPs) between older women classified as having a low or high muscle mass on the basis of SMI and %SMM_r_ indices, and (2) assess the predictive power of GPS_DD_ on skeletal muscle phenotypes related to the upper and lower limb, specifically muscle size and strength measures.

## 2. Methods

### 2.1. Participants

Three-hundred 60- to 91-year-old Caucasian women (70.7 ± 5.7 years, 66.3 ± 11.3 kg, 1.60 ± 0.06 m; mean ± SD) were recruited in the current study from the University of the Third Age (U3A), who were socially engaged in different recreational activities. Some of the participants were recruited via word-of-mouth between participants. The participants reported no symptoms of cardiovascular, muscular, and bone diseases that could interfere with daily activities. All study protocols were in accordance with the guidelines of the Declaration of Helsinki and approved by the local ethics committee of Manchester Metropolitan University. Participants provided written consent prior to involvement in this study.

### 2.2. Muscle Mass, Size, and Strength Related Phenotypes

Skeletal muscle mass (SMM) was estimated using bio-impedance analysis (BIA) (Model 1500; Bodystat, Douglas, Isle of Man, UK) validated for use in a Caucasian population [32] and then used to calculate the skeletal muscle index (SMI) as SMM/height^2^ and %SMM_r_ as 100% × SMM/body mass.

Biceps brachii thickness and vastus lateralis muscle anatomical cross-sectional area (VL_ACSA_) were measured with B-mode ultrasound (My LabTwice, Esaote Biomedical, Genoa, Italy). The measurements were performed at 60% length of humerus (measured from proximal end) for biceps brachii thickness, and at 50% of vastus lateralis muscle length for VL_ACSA_. Intraclass correlation coefficient (*ICC*), based on measurements from 6 participants, for biceps brachii thickness and VL_ACSA_, was high in our population (*ICC* = 0.98 for biceps brachii thickness, *ICC* = 0.99 for VL_ACSA_) [33]. The detailed procedure for obtaining the biceps brachii thickness and VL_ACSA_ using ultrasonography has been described previously [33].

Handgrip strength (HGS) was calculated as the highest value of six trials (three trials each with the left and right hand) using a digital load cell handgrip dynamometer (JAMAR plus, JLW Instruments, Chicago, IL, USA) [31]. The reliability of assessment of HGS is high (*ICC* = 0.99) [34].

A custom-built dynamometer with participants sitting and maintaining a knee angle at 120° extension (180° is equivalent to straight position) was used to assess the knee extension torque (MVC_KE_) of the dominant leg through a calibrated load cell (Zemic, EtenLeur, The Netherlands). Three trials were completed, and the highest force value was recorded and converted to torque (N·m) as

MVC_KE_ = Force × distance from rotation point of dynamometer to ankle strap × Cos 30°

Similarly, the same dynamometer was used to assess elbow flexion torque (MVC_EF_) with participants seated and elbow flexed at 60° (0° is a straight position). Three trials were completed, and the highest force was recorded and subsequently the output was converted to torque (N·m) as

MVC_EF_ = Force × Radius length × Cos 30°

*ICC* for measuring both MVC_EF_ (*ICC* = 0.95) and MVC_KE_ (*ICC* = 0.96) were reported as high with this method [33].

### 2.3. Pre-Sarcopenia/Low Muscle Mass Assessment

Using SMI and %SMM_r_, participants were separated into two groups, either “high muscle mass” or “low muscle mass”, on the basis of pre-sarcopenic thresholds defined in our previous study and others [31]. Individuals with SMI < 6.76 kg/m^2^ were defined as pre-sarcopenic and allocated to the low muscle mass group [21], and with the %SMM_r_ approach, individuals with %SMM_r_ < 22.1% were defined as pre-sarcopenic and allocated to the low muscle mass group [20].

### 2.4. SNPs Selection, DNA Extraction, and Genotyping

Twenty-four SNPs were chosen on the basis of their previous association with skeletal muscle mass, or similar, phenotypes (Table S1). Participants were asked to provide either a venous blood (collected from a superficial forearm vein in EDTA tubes and stored at −20 °C) or saliva (in DNA Saliva kits (Oragene^®^DNA, OG-500, Ottawa, ON, Canada) and stored at room temperature) sample. Genomic DNA was extracted using the Qiagen DNA Blood Mini kit (Qiagen, Crawley, UK).

An EP1 Fluidigm system was used for genotyping as per the manufacturer’s instructions. All samples were analysed in duplicate to minimise the occurrence of genotyping errors [35]. When duplicate samples did not agree (≈1%), they were analysed again using a StepOnePlus instrument (Applied Biosystems, Paisley, UK). The detailed process for genotyping the samples has been explained in our previous paper [31].

### 2.5. Statistical Analysis

All statistical analyses were performed in IBM SPSS Version 27.0, and statistical significance was set at *p* < 0.05. The assessment of Hardy–Weinberg equilibrium (HWE) for all SNPs was performed using the chi-squared test. Then SNPs were examined for linkage disequilibrium (LD), checking for the heterozygotes and homozygotes similarity between the associated SNPs. For two SNPs (*PTK2* rs7460 and *PTK2* rs7843014), there was high LD between the SNPs (LD > 0.8), and only *PTK2* rs7460 was retained for further analyses. GS was assigned to each genotype for all remaining SNPs (*n* = 23), as described previously [22], where the favourable (predisposing) allele—on the basis of previous literature, in the instance of insufficient evidence within the literature using the present dataset—was allocated a score of 1 and least favourable allele as 0; thus, the favourable homozygous genotype was scored 2, heterozygote as 1, and least favourable homozygous genotype as 0 (Table S1). Where there was insufficient evidence in the literature to determine the favourable allele, we coded the direction for allocating a GS on the basis of the β-coefficient as tested by regression (with HGS (a commonly assessed muscle phenotype) as the dependent variable and SNP coding value as the independent variable). If there was a positive β-coefficient value, the scoring of genotypes was retained, while for a negative β-coefficient value, the scoring was reversed. GPS_TOTAL_ of the participants was calculated as the sum of all 23 GSs (Table S1). An independent samples t-test was performed to identify the differences in GPS_TOTAL_ between high muscle mass and low muscle mass groups, using both the %SMM_r_ threshold (%SMM_r_ < 22.1%) [20] and SMI threshold (SMI < 6.76 kg/m^2^) [21].

To establish a GPS_DD_, a backward linear regression with age and individual SNP GSs included in the model was performed to identify the SNPs associated with investigated skeletal muscle phenotypes (biceps brachii thickness, VL_ACSA_, HGS, MVC_EF_, and MVC_KE_). The significance levels for entry and exit in the model were 0.1 and 0.05, respectively. In the instance when the β coefficient was negative, the scoring of the predisposing allele was reversed from the initial scoring. Then, a GPS was calculated for each phenotype by summing GSs of only those SNPs associated with the selected skeletal muscle phenotype in the backward linear regression (*p* < 0.1) as described previously [29]. Finally, linear regressions were carried out with GPS and age as independent variables, and skeletal muscle phenotypes as dependent variables.

For each phenotype, GPSs were grouped according to the number of predisposing alleles possessed, and the mean value was calculated for each group [28]. When a group had few (≤17, <6.0%) participants, it was merged with the group with the nearest number of predisposing alleles.

## 3. Results

Participant characteristics are presented in Table 1. Those in the low muscle mass group according to the SMI threshold (SMI < 6.76 kg/m^2^) had lower %SMM_r_ (−7.4%, *p* < 0.001), biceps brachii thickness (−4.9%, *p* = 0.016), VL_ACSA_ (−12.5%, *p* < 0.001), HGS (−6.1%, *p* = 0.003), MVC_EF_ (−9.5%, *p* < 0.001), and MVC_KE_ (−8.6%, *p* = 0.022) than the high muscle mass group. Similarly, the low muscle mass group based on the %SMM_r_ threshold (%SMM_r_ < 22.1%) had lower SMI (−9.5%, *p* < 0.001), SMM_r_ (−24.8%, *p* < 0.001), body mass (−16.9%, *p* < 0.001), and MVC_KE_ (−11.3%, *p* = 0.041), and higher BMI (16.8%, *p* < 0.001) than the high muscle mass group.

All the SNPs selected were in HWE (*p* > 0.05).

### 3.1. Total Genotype Predisposition Score and Pre-Sarcopenia/Low Muscle Mass

There was no significant difference in GPS_TOTAL_ between low muscle mass and high muscle mass groups using the SMI (*t* (298) = −0.074, *p* = 0.941) or %SMM_r_ (*t* (298) = −1.351, *p* = 0.178) to classify low muscle mass.

### 3.2. Data-Driven GPS and Skeletal Muscle Phenotypes

The GPS_DD_ model explained 1.7% of the variance in biceps brachii thickness, 12.5% in VL_ACSA_, 19.0% in HGS, 8.2% in MVC_EF_, and 9.6% in MVC_KE_ (Table 2). On the basis of the backward regression analysis, 13 out of 23 SNPs were found to be associated with at least one of the phenotypes under investigation. *HIF1A* rs11549465 was associated with four out of five investigated skeletal muscle phenotypes and, in descending number of phenotype associations, *ACE* rs4341 with three; *PTK2* rs7460 and *CNTFR* rs2070802 with two; *MTHFR* rs17421511, *ACVR1B* rs10783485, *CNTF* rs1800169, *MTHFR* rs1801131, *MTHFR* rs1537516, *TRHR* rs7832552, *MSTN* rs1805086, *COL1A1* rs1800012, and *FTO* rs9939609 with one phenotype (Table 2). Similarly, HGS was associated with nine SNPs, VL_ACSA_ with four, MVC_EF_ with three, and biceps brachii thickness and MVC_KE_ each with two (Table 2).

**Figure 1:**
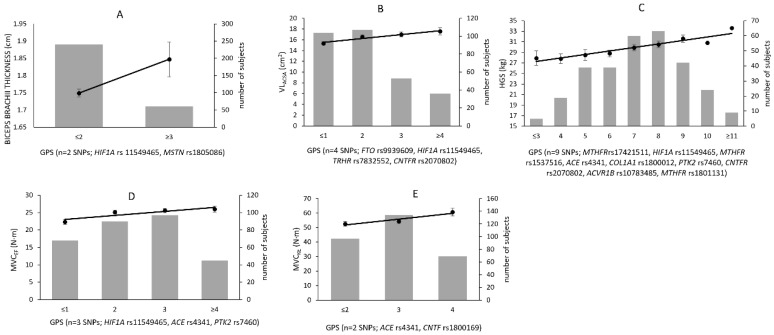
Genetic predisposition score and muscle-related phenotype measures. Participant frequency distribution (bars) and GPS (line) for (**A**) biceps brachii thickness, (**B**) VL_ACSA_ (vastus lateralis anatomical cross-sectional area), (**C**) HGS (hand grip strength), (**D**) MVC_EF_ (elbow flexion maximum voluntary contraction), and (**E**) MVC_KE_ (knee extension maximum voluntary contraction). Dot and error bar represent mean and standard error of the mean, respectively.

There was an increment in most of the muscle size and muscle strength phenotypes in older women according to each additional predisposing allele (Figure 1). For example, subjects possessing ≤1 predisposing allele for VL_ACSA_ had 15.3 ± 0.3 cm^2^ area, those possessing two had 16.5 ± 0.4 cm^2^, those with three had 17.0 ± 0.4 cm^2^, and those possessing ≥4 had 17.6 ± 0.6 cm^2^ (Figure 1 B). Similarly, those possessing ≤1 predisposing allele for MVC_EF_ had 22.3 ± 0.7 N·m, those with two had 25.2 ± 0.6 N·m, those with three had 25.6 ± 0.6 N·m, and those possessing ≥4 had 26.0 ± 0.8 N·m (values are mean ± SE) (Figure 1D).

## 4. Discussion

The present study observed that GPS_TOTAL_, containing 23 SNPs, did not differ between low and high muscle mass groups of older women. We report that a GPS_DD_ model was successful in explaining 2–19% of the observed variance in muscle size and strength phenotypes. The GPS_DD_ model showed that 13 of the 23 selected candidate gene variants were associated with muscle phenotypes, and *HIF1A* rs11549465 was associated with four phenotypes. These results imply that older women possessing fewer predisposing alleles for skeletal muscle size/strength might be more susceptible to early muscle loss, limiting their ability to maintain independence in later life.

Previously, applying a single candidate gene approach, we reported an association of four gene variants (*FTO* rs9939609, *TRHR* rs7832552, *NOS3* rs1799983, and *ESR1* rs4870044) with muscle mass in this same population [31]. However, after combining muscular phenotype-driven SNPs, there was no difference in GPS_TOTAL_ between low and high muscle mass groups (defined on the basis of thresholds of %SMM_r_ and SMI), which aligns with other studies using other polygenic approaches with muscle phenotypes or athletic status [27,36]. Perhaps this unexpected observation is attributable to the fact that a positive score for one SNP may be cancelled by the negative score of another SNP. In addition, the number of SNPs considered in the present study is relatively small compared to what is possible in principle, and we are aware that muscle phenotypes are also very likely to depend on many other gene variants not included in the current study. Genome wide association studies might be useful in identifying more SNPs that are associated with skeletal muscle phenotypes, but the technique is more expensive and requires a larger sample.

The associations between GPS_DD_ and muscle size and strength measures imply that having more favourable alleles, and thus higher GPS score, is beneficial for a larger muscle size and a greater muscle strength. For instance, our study shows that with an increment of one predisposing allele, older women can expect approximate increases in biceps brachii thickness of 0.10 cm, VL_ACSA_ of 0.71 cm^2^, HGS of 0.7 kg, MVC_EF_ of 1.1 N·m, and MVC_KE_ of 3.7 N·m. In coronary artery disease patients (age 60.5 ± 9.6 years), previous use of a GPS_DD_ model demonstrated a comparable association between GPS and increment in rectus femoris diameter after five months of aerobic exercise training [14]. In another way, our study showed that having a low number of predisposing alleles results in small muscle size and low muscle strength, and therefore elderly women with those conditions may lose independence earlier than those with more predisposing alleles for muscle size and strength-related phenotypes. We do, however, acknowledge that different muscles experience differing rates of muscle loss with ageing, and the genetic influence on different muscle groups may vary. It is plausible, therefore, that if axial muscle groups, for example, were considered in the present study, the polygenic profiling may be different from what has been observed in the muscle size and related strength parameters of the selected appendicular muscles in the current study. Our study shows that a regression model that includes GPS and age could explain up to 19% observed variance for HGS in the present older women.

The GPS_DD_ model used in the present study is similar to that of previous studies that investigated the genetic influence on peak VO_2_ [28]; muscle size, muscle strength, and trainability [14]; VO_2_ max training response [37]; and knee extension strength [27,29] in different groups and populations. The variance explained by GPS and age for the skeletal muscle phenotypes in the present study was, however, lower than that observed previously in people ranging from 19 to 73 years of age (for instance, 42.3% variance was explained for knee extension [27]). It should be noted that, apart from the GPS, age was the only additional independent variable used in the present study, while a previous study included age, sex, energy expenditure during sports, and protein intake [27]. In the current study, *HIF1A* rs11549465 was associated with most of the phenotypes, which could be possibly explained by the biological role of HIF1 protein, specifically affecting the transcriptional level of genes involved in cellular proliferation [38]. Overall, the SNPs associated with skeletal muscle phenotypes in the present study are consistent with previous studies showing associations with similar muscle size, strength, and other performance-related phenotypes [39,40,41,42,43,44,45,46,47,48,49,50,51,52,53].

### Limitations

The GPS_DD_ did not consider the possibility that some of the heterozygotes may show complete dominance or over-dominance, because more data regarding each SNP are required to make that refinement. Moreover, the GPS we utilised attributed equal explanatory weight to every SNP, which is unlikely to be accurate—again, more data are required to be able to adjust this accordingly. Furthermore, the analysis did not directly consider potential gene–gene interactions, although the regression approach we used should exclude a SNP if its association with a phenotype is not independent from other such SNP-phenotype associations in the model. In addition, the GPS was constructed and applied to the same dataset, and cross-validation of the observed results is required in another independent older population to evaluate the true (probably lower) proportion of variability explained. The polygenic models used in the current study were based on the selection of SNPs from our previous data [30,31] and the most relevant extant data at the time of analysis [10,54,55]. As with the nature of genotype–phenotype association studies, new candidate genes and SNPS are identified regularly, such that recent research [56,57] have identified new SNPs (e.g., *VCAN* [58]) and some of the same candidate genes that we have chosen (e.g., *FTO*) in the present study, that would form the basis of future genotype–phenotype association studies. The validity of the novel associations identified in our present polygenic models remains valid, with the potential for stronger associations to be found in future research as more candidate genes are identified.

## 5. Conclusions

Although there was no difference in GPS_TOTAL_ between the low and high muscle mass group, the GPS_DD_ model explained up to 19% of the observed muscle phenotype variance, in older women. We conclude that there is a polygenic association with skeletal muscle phenotypes, which needs further investigation, and may help in targeting appropriate interventions for the maintenance of muscle function and thus independence among older adults.

## Figures and Tables

**Table 1:** Characteristics of all participants and according to pre-sarcopenia grouping.

		SMI Threshold		%SMM_r_ Threshold	
	All (*n* = 300)	Low (*n* = 181)	High (*n* = 119)	Low (*n* = 41)	High (*n* = 259)
Age (years)	70.7 ± 5.7	71.0 ± 5.2	70.3 ± 6.3	71.8 ± 5.8	70.6 ± 5.6
Body mass (kg)	66.3 ± 11.3	63.3 ± 9.2	70.8 ± 12.6	77.6 ± 13.3 *	64.5 ± 9.8
Height (kg/m^2^)	1.60 ± 0.06	1.60 ± 0.06	1.59 ± 0.05	1.60 ± 0.05	1.60 ± 0.06
BMI (kg/m^2^)	25.9 ± 4.2	24.6 ± 3.2 *	27.8 ± 4.6	30.3 ± 5.5 *	25.2 ± 3.4
SMI (kg/m^2^)	6.56 ± 0.82	6.04 ± 0.51 *	7.34 ± 0.53	6.01 ± 0.95 *	6.64 ± 0.76
%SMM_r_	25.7 ± 3.8	24.9 ± 3.3	26.9 ± 4.2	20.0 ± 1.3	26.6 ± 3.3
BB thickness (cm)	1.77 ± 0.32	1.73 ± 0.32 *	1.82 ± 0.31	1.85 ± 0.36	1.76 ± 0.31
VL_ACSA_ (cm^2^)	16.3 ± 3.4	15.4 ± 3.1 *	17.6 ± 3.3	16.9 ± 3.9	16.2 ± 3.3
HGS (kg)	30.0 ± 5.0	29.2 ± 4.3 *	31.1 ± 5.6	28.7 ± 4.9	30.2 ± 5.0
MVC_EF_ (N·m)	24.8 ± 5.8	23.8 ± 5.5 *	26.3 ± 6.0	23.3 ± 5.0	25.0 ± 5.9
MVC_KE_ (N·m)	55.2 ± 18.3	53.2 ± 17.3 *	58.2 ± 19.3	49.7 ± 19.3 *	56.0 ± 18.0
GPS_TOTAL_	21.5 ± 2.8	21.5 ± 2.7	21.5 ± 2.9	21.0 ± 2.4	21.6 ± 2.8

Abbreviations: BMI, body mass index; SMI, skeletal muscle index; BB, biceps brachii; VL_ACSA,_ vastus lateralis anatomical cross-sectional area; HGS, hand grip strength; MVC_EF_, isometric elbow flexion maximum voluntary contraction; MVC_KE_, isometric knee extension maximum voluntary contraction; GPS_TOTAL_, Total Genotype Predisposition Score. Values are mean ± SD. * indicates the significant difference from high muscle mass group.

**Table 2:** Regression models for GPS_DD_ and skeletal muscle phenotypes including age as an independent variable.

Phenotypes		GPS_DD_	Age	Adj *r*^2^	Associated SNPs (Predisposing Allele)
Biceps brachii thickness (cm)	estimate	0.101	−0.003		*HIF1A* rs11549465 (T), *MSTN* rs1805086 (T)
	*β* value	0.146	−0.058	1.7%	
	partial *r*	0.146	−0.058		
	*p*	0.014	0.327		
VL_ACSA_ (cm^2^)	estimate	0.710	−0.172		*FTO* rs9939609 (A), *HIF1A* rs11549465 (T), *TRHR* rs7832552 (T), *CNTFR* rs2070802 (T)
	*β* value	0.251	−0.287	12.5%	
	partial *r*	0.258	−0.293		
	*p*	<0.001	<0.001		
HGS (kg)	estimate	0.685	−0.323		*MTHFR* rs17421511 (A), *HIF1A* rs11549465 (T), *MTHFR* rs1537516 (A), *ACE* rs4341 (G), *COL1A1* rs1800012 (A), *PTK2* rs7460 (A), *CNTFR* rs2070802 (T), *ACVR1B* rs10783485 (G), *MTHFR* rs1801131 (T)
	*β* value	0.260	−0.368	19.0%	
	partial *r*	0.278	−0.380		
	*p*	<0.001	<0.001		
MVC_EF_ (N·m)	estimate	1.066	−0.220		*HIF1A* rs11549465 (T), *ACE* rs4341 (G), *PTK2* rs7460 (A)
	*β* value	0.203	−0.213	8.2%	
	partial *r*	0.208	−0.218		
	*p*	<0.001	<0.001		
MVC_KE_ (N·m)	estimate	3.743	−0.803		*ACE* rs4341 (G)*, CNTF* rs1800169 (G)
	*β* value	0.172	−0.258	9.6%	
	partial *r*	0.178	−0.252		
	*p*	0.002	<0.001		

Abbreviations: GPS_DD_, data-driven genotype predisposition score; VL_ACSA_, vastus lateralis anatomical cross-sectional area; HGS, handgrip strength; MVC_EF_, maximum voluntary contraction—elbow flexion; MVC_KE_, maximum voluntary contraction—knee extension.

## Data Availability

The data used in the present study are available from reasonable request from corresponding author.

## Supplementary Materials

The following supporting information can be downloaded at https://www.mdpi.com/article/10.3390/genes13060982/s1. Table S1: Previous associations of single nucleotide polymorphisms with body composition/muscle-related phenotypes/performance (references [59,60,61,62,63,64,65,66,67,68,69,70,71,72,73,74,75,76,77,78,79,80,81,82,83,84] are cited in Supplementary Materials).
