# Supplementary material for: Polygenic Models Partially Predict Muscle Size and Strength but Not Low Muscle Mass in Older Women
Source: Genes (Basel). 2022 May 30;13(6):982. doi: 10.3390/genes13060982 (PMC9223182; doi:10.3390/genes13060982)
Supplement: Supplementary file 1 [file genes-13-00982-s001.zip › genes-1701725-supplementary.pdf]

Supplementary Tables

Table S1. *Previous associations of single nucleotide polymorphisms with body composition/muscle-related phenotypes/performance.*

Single nucleotide polymorphisms	Phenotype	Previous literature for selection of SNPs and the outcome measures	Score allocation for genotype in the present study
ACTN3 rs1815739	Muscle size<br><br>Knee extension torque<br><br>Sarcopenia<br><br>Elite sprint athlete status	XX genotype had lower mid-thigh area than RX/RR (Zempo et al., 2010) [37]<br><br>XX genotype had lower knee torque than RR/RX. (Walsh et al., 2008) [33]<br><br>XX genotype at higher risk of sarcopenia (Cho et al., 2017) [6]<br><br>RR genotype overrepresented in elite sprinter group versus controls (Chen et al., 2020) [5]	RR=2, RX=1, XX=0
ACE rs4341 (I/D)	Lean mass	D allele favourable for higher lean mass (Charbonneau et al., 2008) [4]	DD=2, ID=1, II=0
CNTF rs1800169	Knee flexion<br><br>Knee extension<br><br>Handgrip strength	A-allele carriers weaker than GG homozygotes in middle aged women (De Mars et al., 2007) [9]<br><br>G-allele favourable for knee strength among elderly (He et al., 2020) [16]<br><br>AA homozygotes had 3.8 kg weaker handgrip strength than G-allele carriers (Arking et al., 2006)[2]	AA=0, AG=1, GG=2
CNTFR rs2070802	Knee extension and flexion	T-allele carriers could produce greater torque (De Mars et al., 2007) [9]	TT=2, AT=1, AA=0
ESR1 rs4870044<br><br>ESR1 rs1999805	Sarcopenia	T-allele carriers had higher risk of sarcopenia (Khanal et al., 2020) [19]	CC=2, CT=1, TT=0<br><br>AA=2, AG=1, GG=0, (based on beta coefficient)
FTO rs9939609	Muscle area	AA homozygotes had a larger vastus lateralis muscle area (Khanal et al., 2020) [19]	AA=2, AT=1, TT=0
HIF1A rs11549465	Oxygen consumption capacity<br><br>Strength/power athlete status	TT associated with higher $V0_{2max}$ among elderly (Prior et al., 2003) [23]<br><br>Overrepresentation of TT genotype in weightlifters, wrestlers and power-oriented athletes compared to controls (Ahmetov et al., 2008 [1], Drozdovska et al., 2013 [10])	TT=2, CT=1, CC=0
ID3 rs11574	Obesity-related indices	A-allele associated with increment in BMI and fat mass (Svendstrup et al., 2018) [30]	TT=0, CT=1, CC=2 (based on beta coefficient)
IGF1 rs35767	Body composition	GG homozygotes had greater total fat but lower lean and muscle mass (Kostek et al., 2010) [20]	AA=2, AG=1, GG=0
IL6 rs1800795	Elite athlete status	Overrepresentation of G- allele among weightlifters, jumpers, and elite athletes (Ruiz et al., 2010 [25], Cenikli et al., 2016 [3])	GG=2, GC=1, CC=0

MTHFR<br>rs1801131<br>rs1537516<br>rs17421511	Strength/sprint athlete status<br><br>Maximal rate of oxygen consumption	Overrepresentation of rs1801131 C-allele among strength and sprint athletes (Zarebska et al., 2014) [36]<br><br>rs1801131 C-allele carriers had greater improvement in VO_{2max} during training (Cięszczyk et al., 2016) [7]	MTHFR rs1801131 (GG=2, GT=1, TT=0)<br><br>MTHFR rs1537516 (AA=0, AG=1, GG=2) (based on beta coefficient)<br><br>*MTHFR* rs17421511 (AA=2, AG=1, GG=0) (based on beta coefficient)
PTK2<br>rs7843014,<br>rs7460	Specific force	AA homozygotes had greater vastus lateralis specific force in healthy population (Erskine et al., 2012 [12], Stebbings et al., 2017 [29])	*PTK2* rs7843014 (AA=2, AC=1, CC=0)<br><br>*PTK2* rs7460 (AA=2, AT=1, TT=0)
TRHR rs7832552	Lean body mass<br><br>Sarcopenia	T-allele favoured greater lean body mass (Liu et al., 2009 [21], Lunardi et al., 2013 [22])<br><br>C-allele carriers at higher risk of sarcopenia than TT homozygotes (Khanal et al., 2020) [19]	TT=2, TC=1, CC=0
TTN rs10497520	Endurance running performance	T-allele carriers had better marathon performance (Stebbings et al., 2018) [28]	TT=2, CT=1, CC=0
VDR rs2228570	Muscle size<br><br>Knee strength	C- allele associated with lower fat-free-mass (Roth et al., 2004) [24]<br><br>C/CC allele/genotype had lower knee strength compared to f-allele carriers (Windelinckx et al., 2007 [34], Hopkinson et al., 2008 [18])	AA=2, AG=1, GG=0
MSTN rs1805086	Muscle strength/muscle size	C- allele associated with lower muscle strength (Seibert et al., 2001 [26], Corsi et al., 2002 [8])<br><br>C- allele associated with lower muscle mass among elderly women (González-Freire et al., 2010) [14]	TT=2, CT=1, CC=0
COL1A1 rs1800012	Muscle strength	A-allele associated with lower handgrip and biceps strength in elderly (Van Pottelbergh et al., 2001 [31])	CC=2, AC=1, AA=0
ACVR1B<br>rs2854464	Muscle strength<br><br>Sprint/power athlete status	A-allele associated with higher knee strength (Windelinckx et al., 2011 [35])<br><br>A-allele overrepresented in sprint and power athletes (Voisin et al., 2016 [32])	GG=0, GA=1, AA=2
ACVR1B<br>rs10783485	Muscle mass	A-allele associated with higher SMM (He et al., 2018 [17])	GG=0, GT=1, TT=2
NOS3 rs1799983	Power athlete status<br><br>Stroke volume<br><br>Sarcopenia	T-allele common among power athletes (Gómez-Gallego et al., 2009 [13], Sessa et al., 2011 [27], Zmijewski et al., 2018 [38], Eider et al., 2014 [11])<br><br>Postmenopausal women with T-allele had higher stroke volume during dynamic exercise (Hand et al., 2006) [15]<br><br>T-allele carriers had higher skeletal muscle mass above sarcopenic threshold (Khanal et al., 2020) [19]	TT=2, CT=1, CC=0

1. Ahmetov, II, Hakimullina, A.M., Lyubaeva, E.V., Vinogradova, O.L. and Rogozkin, V.A. Effect of HIF1A gene polymorphism on human muscle performance. *Bulletin of experimental biology and medicine.* **2008**, *146*, 351–353.
2. Arking, D.E., Fallin, D.M., Fried, L.P., Li, T., Beamer, B.A., Xue, Q.L., Chakravarti, A. and Walston, J. Variation in the ciliary neurotrophic factor gene and muscle strength in older Caucasian women. *Journal of the American Geriatrics Society.* **2006**, *54*, 823–826.
3. Cenikli, A., Nursal, A., Tural, E., Polat, Y., Tasmektepligil, M. and Yigit, S. The Correlation Between Rs1800795 Variant of IL-6 and sports performance among turkish elite athletes. *International Journal of Humanities Social Sciences and Education.* **2016**, *3*, 1–5.
4. Charbonneau, D.E., Hanson, E.D., Ludlow, A.T., Delmonico, M.J., Hurley, B.F. and Roth, S.M. ACE genotype and the muscle hypertrophic and strength responses to strength training. *Medicine and science in sports and exercise.* **2008**, *40*, 677.
5. Chen, W., Zhang, B., Li, Y. and Liang, T. Association Between rs1815739 Polymorphism of ACTN3 Gene and Athletic Ability in Chinese Sprinters. *Journal of Science in Sport and Exercise,* **2020**, *2*, 113–119.
6. Cho, J., Lee, I. and Kang, H. ACTN3 gene and susceptibility to sarcopenia and osteoporotic status in older Korean adults. *BioMed research international.* 2017
7. Cięszczyk, P., Zarębska, A., Jastrzębski, Z., Sawczyn, M., Kozakiewicz-Drobnik, I., Leońska-Duniec, A., Kaczmarczyk, M., Maciejewska-Skrendo, A., Żmijewski, P. and Trybek, G. Does the MTHFR A1298C Polymorphism Modulate the Cardiorespiratory Response to Training? *Journal of human kinetics.* **2016**, 54**,** 43–53.
8. Corsi, A.M., Ferrucci, L., Gozzini, A., Tanini, A. and Brandi, M.L. Myostatin polymorphisms and age-related sarcopenia in the Italian population. *Journal of the American Geriatrics Society.* **2002**, *50*, 1463.
9. De Mars, G., Windelinckx, A., Beunen, G., Delecluse, C., Lefevre, J. and Thomis, M.A.I. Polymorphisms in the CNTF and CNTF receptor genes are associated with muscle strength in men and women. *Journal of Applied Physiology.* **2007**, *102*, 1824–1831.
10. Drozdovska, S., Dosenko, V., Ahmetov, I. and Ilyin, V. The association of gene polymorphisms with athlete status in Ukrainians. *Biology of sport.* **2013**, *30*, 163.
11. Eider, J., Ficek, K., Kaczmarczyk, M., Maciejewska-Karłowska, A., Sawczuk, M. and Cięszczyk, P. Endothelial nitric oxide synthase g894t (rs1799983) gene polymorphism in polish athletes. *Open Life Sciences.* **2014**, *9*, 260–267.
12. Erskine, R.M., Williams, A.G., Jones, D.A., Stewart, C.E. and Degens, H. Do PTK2 gene polymorphisms contribute to the interindividual variability in muscle strength and the response to resistance training? A preliminary report. *Journal of applied physiology.* **2012**, *112*, 1329–1334.
13. Gómez-Gallego, F., Ruiz, J.R., Buxens, A., Artieda, M., Arteta, D., Santiago, C., Rodríguez-Romo, G., Lao, J.I. and Lucia, A. The− 786 T/C polymorphism of the NOS3 gene is associated with elite performance in power sports. *European journal of applied physiology.* **2009**, *107*, 565–569.
14. González-Freire, M., Rodríguez-Romo, G., Santiago, C., Bustamante-ARA, N., Yvert, T., Gómez-Gallego, F., Rexach, J.A.S., Ruiz, J.R. and Lucia, A. The K153R variant in the myostatin gene and sarcopenia at the end of the human lifespan. *Age.* **2010**, *32*, 405–409.
15. Hand, B.D., Mccole, S.D., Brown, M.D., Park, J.J., Ferrell, R.E., Huberty, A., Douglass, L.W. and Hagberg, J.M. NOS3 gene polymorphisms and exercise hemodynamics in postmenopausal women. *International journal of sports medicine.* **2006**, *27*, 951–958.
16. He, L., Khanal, P., Morse, C.I., Williams, A. and Thomis, M. Associations of combined genetic and epigenetic scores with muscle size and muscle strength: A pilot study in older women. *Journal of Cachexia, Sarcopenia and Muscle*. **2020**.
17. He, L., Van Roie, E., Bogaerts, A., Morse, C.I., Delecluse, C., Verschueren, S. and Thomis, M. Genetic predisposition score predicts the increases of knee strength and muscle mass after one-year exercise in healthy elderly. *Experimental gerontology.* **2018**, *111*, 17–26.
18. Hopkinson, N.S., Li, K.W., Kehoe, A., Humphries, S.E., Roughton, M., Moxham, J., Montgomery, H. and Polkey, M.I. Vitamin D receptor genotypes influence quadriceps strength in chronic obstructive pulmonary disease. *The American journal of clinical nutrition.* **2008**, *87*, 385–390.
19. Khanal, P., He, L., Stebbings, G., Onambele-Pearson, G.L., Degens, H., Williams, A., Thomis, M. and Morse, C.I. Prevalence and association of single nucleotide polymorphisms with sarcopenia in older women depends on definition. *Scientific reports.* **2020**, *10*, 1–9.
20. Kostek, M.C., Devaney, J.M., Gordish-Dressman, H., Harris, T.B., Thompson, P.D., Clarkson, P.M., Angelopoulos, T.J., Gordon, P.M., Moyna, N.M. and Pescatello, L.S. A polymorphism near IGF1 is associated with body composition and muscle function in women from the Health, Aging, and Body Composition Study. *European journal of applied physiology.* **2010**, *110*, 315–324.
21. Liu, X.-G., Tan, L.-J., Lei, S.-F., Liu, Y.-J., Shen, H., Wang, L., Yan, H., Guo, Y.-F., Xiong, D.-H. and Chen, X.-D. Genome-wide association and replication studies identified TRHR as an important gene for lean body mass. *The American Journal of Human Genetics.* **2009**, *84*, 418–423.

22. Lunardi, C.C., Lima, R.M., Pereira, R.W., Leite, T.K.M., Siqueira, A.B.M. and Oliveira, R.J. Association between polymorphisms in the TRHR gene, fat-free mass, and muscle strength in older women. *Age.* **2013**, *35*, 2477–2483.
23. Prior, S.J., Hagberg, J.M., Phares, D.A., Brown, M.D., Fairfull, L., Ferrell, R.E. and Roth, S.M. Sequence variation in hypoxia-inducible factor 1α (HIF1A): Association with maximal oxygen consumption. *Physiological genomics.* **2003**, *15*, 20–26.
24. Roth, S.M., Zmuda, J.M., Cauley, J.A., Shea, P.R. and Ferrell, R.E. Vitamin D receptor genotype is associated with fat-free mass and sarcopenia in elderly men. *The Journals of Gerontology Series A: Biological Sciences and Medical Sciences.* **2004**, *59*, B10–B15.
25. Ruiz, J.R., Buxens, A., Artieda, M., Arteta, D., Santiago, C., Rodríguez-Romo, G., Lao, J.I., Gómez-Gallego, F. and Lucia, A. The− 174 G/C polymorphism of the IL6 gene is associated with elite power performance. *Journal of science and medicine in sport.* **2010**, *13*, 549–553.
26. Seibert, M.J., Xue, Q.L., Fried, L.P. and Walston, J.D. Polymorphic variation in the human myostatin (GDF-8) gene and association with strength measures in the women's health and aging study II cohort. *Journal of the American Geriatrics Society.* **2001**, *49*, 1093–1096.
27. Sessa, F., Chetta, M., Petito, A., Franzetti, M., Bafunno, V., Pisanelli, D., Sarno, M., Iuso, S. and Margaglione, M. Gene polymorphisms and sport attitude in Italian athletes. *Genetic testing and molecular biomarkers.* **2011**, *15*, 285–290.
28. Stebbings, G.K., Williams, A.G., Herbert, A.J., Lockey, S.J., Heffernan, S.M., Erskine, R.M., Morse, C.I. and DAY, S.H. TTN genotype is associated with fascicle length and marathon running performance. *Scandinavian journal of medicine & science in sports.* **2018**, *28*, 400–406.
29. Stebbings, G.K., Williams, A.G., Morse, C.I. and Day, S.H. Polymorphisms in PTK2 are associated with skeletal muscle specific force: An independent replication study. *European journal of applied physiology.* **2017**, *117*, 713–720.
30. Svendstrup, M., Appel, E.V.R., Sandholt, C.H., Ahluwalia, T.S., Ängquist, L.H., Thuesen, B.H., Jørgensen, M.E., Pedersen, O., Grarup, N. and Hansen, T. Prospective studies exploring the possible impact of an ID3 polymorphism on changes in obesity measures. *Obesity.* **2018**, *26*, 747–754.
31. Van Pottelbergh, I., Goemaere, S., Nuytinck, L., De Paepe, A. and Kaufman, J.M. Association of the type I collagen alpha1 Sp1 polymorphism, bone density and upper limb muscle strength in community-dwelling elderly men. *Osteoporosis International.* **2001**, *12*, 895–901.
32. Voisin, S., Guilherme, J.P.F.L., Yan, X., Pushkarev, V.P., Cieszczyk, P., Massidda, M., Calò, C.M., Dyatlov, D.A., Kolupaev, V.A. and Pushkareva, Y.E. ACVR1B rs2854464 is associated with sprint/power athletic status in a large cohort of Europeans but not Brazilians. *PLoS ONE.* **2016**, *11*, e0156316.
33. Walsh, S., Liu, D., Metter, E.J., Ferrucci, L. and Roth, S.M. ACTN3 genotype is associated with muscle phenotypes in women across the adult age span. *Journal of Applied Physiology.* **2008**, *105*, 1486–1491.
34. Windelinckx, A., De Mars, G., Beunen, G., Aerssens, J., Delecluse, C., Lefevre, J. and Thomis, M.A.I. Polymorphisms in the vitamin D receptor gene are associated with muscle strength in men and women. *Osteoporosis International.* **2007**, *18*, 1235–1242.
35. Windelinckx, A., De Mars, G., Huygens, W., Peeters, M.W., Vincent, B., Wijmenga, C., Lambrechts, D., Delecluse, C., Roth, S.M. and Metter, E.J. Comprehensive fine mapping of chr12q12-14 and follow-up replication identify activin receptor 1B (ACVR1B) as a muscle strength gene. *European Journal of Human Genetics.* **2011**, *19*, 208.
36. Zarebska, A., Ahmetov, I.I., Sawczyn, S., Weiner, A.S., Kaczmarczyk, M., Ficek, K., Maciejewska-Karlowska, A., Sawczuk, M., Leonska-Duniec, A. and Klocek, T. Association of the MTHFR 1298A> C (rs1801131) polymorphism with speed and strength sports in Russian and Polish athletes. *Journal of sports sciences.* **2014**, *32*, 375–382.
37. Zempo, H., Tanabe, K., Murakami, H., Iemitsu, M., Maeda, S. and Kuno, S. ACTN3 polymorphism affects thigh muscle area. *International journal of sports medicine.* **2010**, *31*, 138–142.
38. Zmijewski, P., Cieszczyk, P. and Ahmetov, II. The NOS3 G894T (rs1799983) and-786T/C (rs2070744) polymorphisms are associated with elite swimmer status. *Biol Sport.* **2018**, *35*, 313–319.
